# A Rolling Bearing Fault Diagnosis Method Based on PSO-Optimized FHN Stochastic Resonance

**DOI:** 10.3390/s26082408

**Published:** 2026-04-14

**Authors:** Ziqiao Wang, Yongqi Chen, Qinge Dai, Jun Wang, Jiqiang Hu, Lingqiang Wu, Rui Qin

**Affiliations:** 1 College of Science and Technology, Ningbo University, Cixi 315300, China; 2 Ningbo Donghuang Bearing Co., Ltd., Cixi 315300, China

**Keywords:** bearing fault diagnosis, stochastic resonance, FHN-SR, particle swarm optimization, weak feature extraction, noise robustness

## Abstract

Early bearing faults are often difficult to identify because their characteristic components are weak and easily masked by strong interference. To improve weak-fault feature extraction, this paper proposes a particle-swarm-optimization-based FitzHugh–Nagumo stochastic resonance (FHN-SR) method for bearing vibration signals. The raw signal is first preprocessed by de-meaning, Hilbert envelope demodulation, and standardization to construct a stable stochastic resonance (SR) input. Then, the key model parameters are adaptively optimized by maximizing the output signal-to-noise ratio around the target fault characteristic frequency. To evaluate the proposed method comprehensively, comparisons are carried out with classical SR, underdamped bistable stochastic resonance (UBSR), and a Fast-Kurtogram-based envelope-analysis scheme. Experimental validation is performed on three fault cases, including the rolling element fault case from the Case Western Reserve University (CWRU) dataset and the inner-race and outer-race fault cases from the Machinery Comprehensive Diagnostics Simulator (MCDS) platform. The results show that FHN-SR produces a clearer concentration of fault-related energy and achieves a higher output signal-to-noise ratio (SNR) than the compared methods in most cases. In particular, under degraded noise conditions, FHN-SR maintains more stable enhancement performance, indicating stronger robustness to interference. These results demonstrate that the proposed method provides an effective approach for extracting weak bearing fault features under complex noise backgrounds.

## 1. Introduction

Rolling element bearings are among the most critical components in rotating machinery, and a large proportion of system failures can be attributed to bearing defects [1,2]. Therefore, condition monitoring for rotating machinery is essential, and the high failure rate of rolling element bearings motivates the development of online diagnostic systems that can work reliably under varying operating conditions [3,4]. Early fault detection is particularly important because it can reduce costly downtime and help prevent severe malfunctioning or even catastrophic failures. However, practical vibration measurements are often heavily contaminated by noise, and conventional spectral analysis may lose fault-related peaks when they are buried by strong random components, which makes early-stage faults difficult to detect [5,6]. Consequently, detecting incipient bearing faults remains challenging, especially at the initial stage where defects must be identified in time to avoid machinery malfunctioning.

Envelope demodulation is a classic approach for rolling-bearing fault diagnosis and is often used to cope with non-stationary, weak and nonlinear vibration signatures. A typical pipeline selects a resonance/carrier band, applies band-pass filtering, then performs the Hilbert transform to obtain the envelope and inspects the envelope spectrum for fault features [7]. To improve signal-to-noise ratio (SNR) and mitigate spectral artifacts, Wang and Liu suggest selecting a band with large power-spectrum differences between healthy and faulty signals and using zero-padding to avoid wrapping effects before Fourier operations [8]. Nevertheless, identifying the optimal demodulation band is often empirical in practice, which limits the robustness of envelope-based diagnosis in engineering scenarios. To address this, Eddine and Slimane combine Fast Kurtogram with Hilbert envelope analysis and locate an optimal demodulation band via a 2-D kurtosis map for defect detection [9]. Meanwhile, Sheen discusses using Morlet wavelets within a Hilbert-transform-based envelope detection framework, aiming to reduce sensitivity to conventional band-pass selection [10]. Since different envelope demodulation implementations may yield different diagnostic outcomes, Bauer et al. provide a comparative study, and Lyons also recommends implementing multiple detectors and choosing the most suitable one for the given signal and sampling rate [11,12]. Selecting the optimal demodulation band is crucial for envelope-based bearing diagnosis. While Kurtogram is widely used, it can be easily misled by noise and random impulses, leading to pseudo-optimal bands [13,14]. To improve robustness, many enhanced band-selection schemes have been proposed (e.g., envelope-spectrum-based kurtosis variants, improved correlated-kurtosis with PSO, OMA-guided resonance selection, and optimization-driven frameworks such as GA), aiming to work better under strong interference and even variable-speed conditions [15,16,17,18].

In addition to envelope-demodulation-based approaches, statistical-feature-based methods have also been widely used in bearing fault diagnosis and degradation assessment. For example, Liu et al. [19] developed a spalling propagation assessment algorithm for ball bearings by combining a spectrum amplitude ratio with statistical features. In their study, 25 time-domain statistical features were calculated, and Pearson correlation coefficient (PCC) was used to determine the effective features for identifying the spalling damage location and level. These results indicate that statistical descriptors can provide useful information for characterizing bearing damage evolution. However, such methods mainly focus on feature-based damage assessment, whereas the influences of noise and other structural vibrations on the diagnostic results were noted to be beyond the scope of that work. Therefore, for early fault diagnosis under strong background noise, methods capable of directly enhancing weak fault-related components remain highly desirable.

Benzi [20] originally introduced stochastic resonance (SR) to explain periodic phenomena in climate systems, highlighting the counterintuitive mechanism that an appropriate level of noise, when coupled with a nonlinear system, can enhance weak periodic signals. Later, Qiao and Lu [21,22] provided comprehensive overviews of SR models and their evolution in machinery and rotating-machine fault detection, laying a roadmap for subsequent model design and evaluation choices. To tackle the practical issue that weak fault signatures are often submerged in heavy noise, He [23] proposed multiscale noise tuning SR for enhanced fault diagnosis in rotating machines, while He and Wang [24] further analyzed how multiscale noise tuning affects SR performance, offering guidance on scale/noise selection in practice. To relax classical constraints and improve adaptability to complex operating conditions, Lu [25] investigated an underdamped, step-varying second-order SR for weak signal detection, whereas Liu [26] developed an adaptive SR within a new nonlinear system to improve bearing diagnosis efficiency. On the potential-structure side, Li [27] explored multi-stable SR for mechanical fault diagnosis, Cui [28] advanced to coupled multi-stable SR for impulsive-feature enhancement, and Li [29] further proposed a high-dimensional space coupled bistable SR, arguing that an “optimal dimension” may maximize output SNR and demonstrating improved anti-noise robustness on bearing signals. To overcome the inherent output saturation of classical bistable SR, Qiao [30] proposed an adaptive unsaturated bistable SR using a piecewise bistable potential, while He [31] introduced an unsaturated piecewise-linear quad-stable SR and optimized its parameters via an adaptive genetic algorithm, also interpreting particle-jumping behavior from the potential-function structure. Meanwhile, Qiao [32] analyzed SR induced by varying potential-well depth and width, reported double resonance peaks, and noted that the output SNR behavior can be sensitive to initial conditions under different stochastic settings—pointing to robustness challenges in real deployments. To reduce manual tuning and prior knowledge requirements, López [33] pointed out that traditional SR often assumes the characteristic fault frequency is known, and then proposed a CHMM and sparse-measure-driven adaptive search framework that can determine the characteristic frequency directly from measured data; importantly, their framework employs FitzHugh–Nagumo stochastic resonance (FHN-SR) as the SR core. Beyond machinery, Ikemoto [34] applied SR to noise-modulated neural networks, and Wu [35] leveraged SR for nonlinearity-enhanced continuous microwave detection, reinforcing SR’s general-purpose weak-signal enhancement capability. Nevertheless, despite progress via multiscale tuning, underdamped second-order designs, coupled/high-dimensional extensions, unsaturated potentials, and adaptive optimization, SR methods can still be constrained by potential-structure design, multi-parameter tuning burdens, and operating-condition sensitivity.

Although SR has shown considerable potential for weak fault-feature enhancement, its practical application to bearing vibration signals remains challenging. Classical SR and many of its variants are often sensitive to parameter settings, and their performance may degrade markedly under strong interference. In addition, different SR models usually involve different dynamic structures and potential functions, making it difficult to assess whether the observed performance gain originates from the resonance mechanism itself or from the specific model configuration. Therefore, a more systematic comparison under a unified framework is still needed, particularly when conventional non-SR signal-processing baselines are also taken into account.

To address these issues, this study develops a PSO-based FHN-SR framework for bearing fault feature enhancement and performs a comparative investigation with classical SR and underdamped bistable stochastic resonance (UBSR) under a unified signal-processing procedure. The vibration signal is preprocessed by de-meaning, Hilbert envelope demodulation, and standardization to construct a stable SR input, and the key model parameters are optimized by maximizing the output SNR around the target characteristic frequency. In addition to the SR-based methods, a Fast-Kurtogram-based envelope-analysis scheme is introduced as a conventional non-SR baseline. The proposed framework is validated using three fault cases, including the rolling element fault case from the Case Western Reserve University (CWRU) dataset and the inner-race and outer-race fault cases from the Machinery Comprehensive Diagnostics Simulator (MCDS) platform. The results show that FHN-SR achieves the most favorable overall enhancement performance, with clearer concentration of fault-related energy and stronger robustness under severe noise conditions.

## 2. Stochastic Resonance Theory

### 2.1. Classical Stochastic Resonance

SR was originally proposed by Benzi [20] in studies of paleoclimate and glaciation. It refers to a nonlinear phenomenon in which a weak periodic signal, although insufficient by itself to induce state transitions in a bistable system, can be enhanced with the assistance of an appropriate level of noise. Under such conditions, the system output may switch between the two stable states at a rate related to the weak periodic excitation, thereby enhancing the target periodic component. This mechanism constitutes the fundamental idea of classical SR, in which a weak periodic signal, a nonlinear bistable system, and noise jointly determine the resonance behavior. The bistable system is governed by the Langevin equation:

(1) $$\frac{dx}{dt}=-U^{\prime }(x)+S(t)+N(t),$$

where x denotes the system state variable, S(t)=Acos(Ωt+ϕ) is the periodic input, and $N(t)=\sqrt{2D}\;\xi (t)$ is additive Gaussian white noise with zero mean. The stochastic resonance potential function is written as

(2) $$U(x)=-\frac{1}{2}ax^{2}+\frac{1}{4}bx^{4},$$

where a>0 and b>0 are the parameters of the bistable potential.

**Figure 1:**
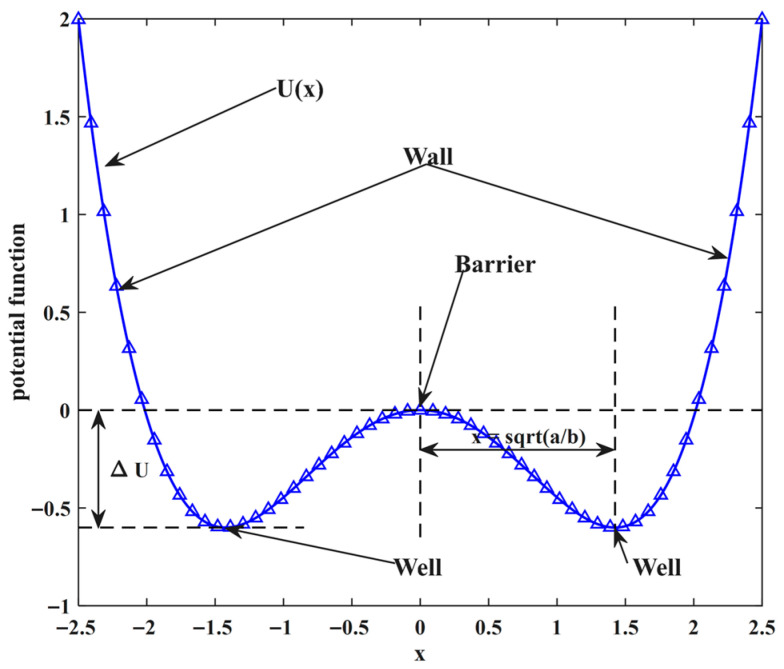
Classical bistable potential function.

By adjusting the system parameters a and b, different potential-well shapes can be obtained. The corresponding barrier height is given by $\Delta U=a^{2}/(4b)$, as illustrated in Figure 1. The potential function U(x) admits two stable equilibria at $x_{m}=\pm \sqrt{a/b}$ and one unstable equilibrium at x=0. Therefore, varying the system parameters enables flexible generation of bistable potential functions with different profiles.

### 2.2. Underdamped Bistable Stochastic Resonance

To enrich the dynamic response of the classical first-order SR model, an UBSR model is considered as a second-order extension. Its governing equation is expressed as

(3) $$\frac{d^{2}x}{dt^{2}}+g\frac{dx}{dt}=a\;x-b\;x^{3}+S\left(t\right)+N\left(t\right),$$

where g is the damping coefficient, and a and b control the bistable potential shape. Compared with classical SR, the introduction of the inertial and damping terms allows the UBSR model to exhibit richer nonlinear dynamics and greater flexibility for weak-fault feature enhancement.

Although classical SR has shown potential in weak-signal enhancement, it remains sensitive to parameter settings and may suffer from insufficient selectivity under strong interference. To overcome these limitations, this study further considers second-order underdamped SR frameworks, including UBSR and FHN-SR, and compares them under a unified signal-processing procedure.

## 3. FHN-SR and Optimization Framework

**Figure 2:**
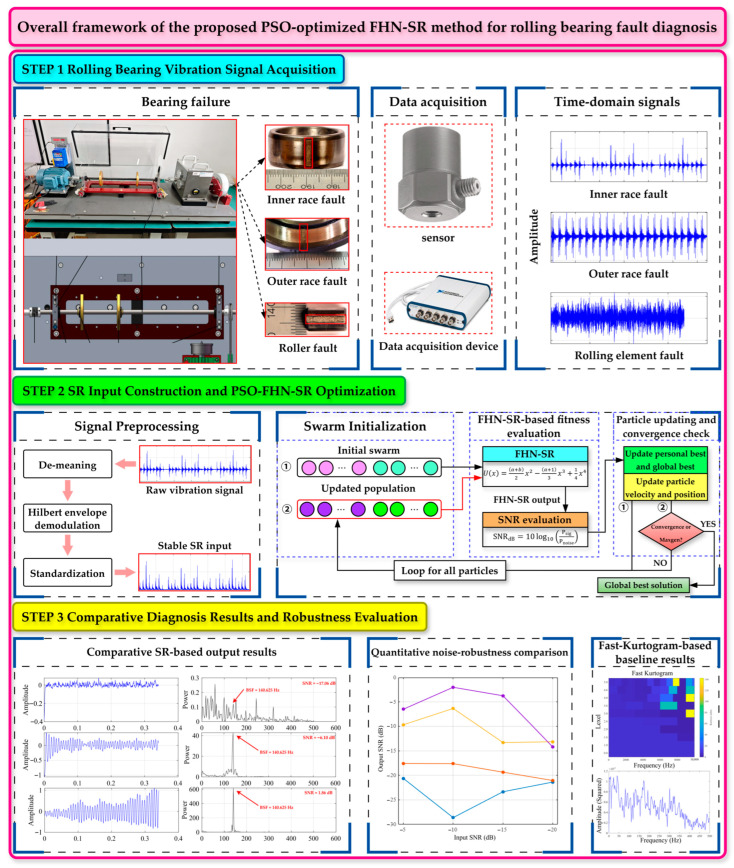
Overall framework of the proposed PSO-optimized FHN-SR method for rolling bearing fault diagnosis.

The purpose of using FHN-SR is to address the following issues: First, traditional stochastic resonance suffers from the small-parameter limitation, i.e., the input amplitude and frequency are typically required to be far less than one, which engineering signals often violate. Second, the conventional bistable SR uses a relatively rigid potential and is highly sensitive to matching. Third, the enhancement capability of a first-order SR method can be insufficient under heavy noise. The flowchart is shown in Figure 2, and the following section details the implementation and explanation of each step.

### 3.1. Signal Preprocessing

Bearing-fault signatures are commonly manifested as resonance modulations excited by impulsive events, while the raw vibration signal often contains structural resonances, background noise, and broadband components unrelated to the fault. To improve the robustness of subsequent SR enhancement and parameter optimization, the input signal is preprocessed through de-meaning, Hilbert envelope demodulation, and standardization.

First, the raw vibration signal x(t) is de-meaned to remove the DC component:

(4) $$x_{0}\left(t\right)=x\left(t\right)-\frac{1}{T}\int_{0}^{T}x\left(t\right)\;dt,$$

Next, the Hilbert transform is used to form the analytic signal and extract the envelope e(t), which demodulates the resonance response and highlights periodic fault information near the characteristic frequency:

(5) $$e\left(t\right)=\left|\;x_{0}\left(t\right)+j\;H\{x_{0}\left(t\right)\}\right|,$$

where H{⋅} denotes the Hilbert transform.

Finally, to reduce the influence of amplitude-scale variations on SR parameter search and to improve numerical stability, the envelope is standardized and used as the SR input u(t):

(6) $$u\left(t\right)=\frac{e\left(t\right)-\mu_{e}}{\sigma_{e}+\varepsilon },$$

where $\mu_{e}$ and $\sigma_{e}$ are the mean and standard deviation of e(t), respectively, and ε is a small constant to avoid division by zero. The resulting u(t) provides a stable and reproducible representation for subsequent SR enhancement and PSO-based optimization.

### 3.2. Underdamped Stochastic Resonance Based on FitzHugh–Nagumo Potential

The SR phenomenon is generally characterized by three fundamental ingredients: (1) a nonlinear system; (2) a weak input signal; and (3) an intrinsic noise source in the system. As noted above, classical SR is subject to the so-called small-parameter limitation, which requires the amplitude and frequency of the periodic signal to be much smaller than unity. In practical applications, however, engineering signals often fail to satisfy this constraint. In this study, an underdamped second-order formulation is adopted to address this issue. The governing equation of the underdamped second-order SR system is given by:

(7) $$\ddot{x}=F\left(x\right)-g\dot{x}+u\left(t\right),$$

where g is the damping coefficient and F(x)=−dU(x)/dx is determined by the adopted potential function. In this study, the FHN-type potential introduced in Ref. [36] is used, i.e.,

(8) $$U\left(x\right)=\frac{\left(a+b\right)}{2}x^{2}-\frac{\left(a+1\right)}{3}x^{3}+\frac{1}{4}x^{4},$$

where a and b are positive parameters controlling the potential shape. Compared with the classical bistable potential, the FHN-type potential provides a more flexible nonlinear structure for adjusting the well shape and the barrier profile, which is beneficial for weak-feature enhancement under complex noise conditions.

**Figure 3:**
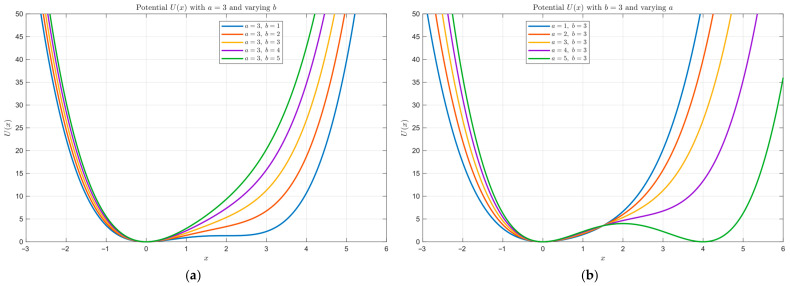
Potential curves: (**a**) a = 3, with b varying from 1 to 5; (**b**) b = 3, with a varying from 1 to 5.

To illustrate the influence of the potential parameters, Figure 3 presents the potential curves obtained by varying b with a=3 and varying a with b=3. It can be observed that changes in a and b lead to clear variations in the profile of the potential function, indicating that the nonlinear restoring characteristics of the system are strongly dependent on the parameter settings. In the subsequent optimization framework, however, the potential parameters are fixed, and the parameter search is focused on the dynamic variables in order to reduce the search dimensionality and improve optimization stability.

The corresponding dynamic equation is numerically solved using the fourth-order Runge–Kutta (RK4) method. RK4 was adopted because the proposed FHN-SR model is a nonlinear second-order dynamical system, for which repeated time-domain integration is required during PSO-based parameter optimization. In this context, numerical accuracy is important because the fitness evaluation is based on the output signal-to-noise ratio, which can be affected by integration error; numerical stability is also important because unstable or overly step-sensitive solutions may interfere with the consistency of parameter search. Meanwhile, computational cost must also be considered, since each particle and each iteration requires repeated integration of the SR model. Compared with lower-order schemes, RK4 provides higher accuracy under a given step size, while remaining simpler and less computationally demanding than more sophisticated adaptive solvers. Therefore, RK4 was selected as a practical compromise between accuracy, stability, and computational efficiency in the present optimization framework.

### 3.3. Particle Swarm Optimization

Since the SR output is obtained via numerical integration, an exhaustive grid search over model parameters is computationally expensive. Therefore, PSO is employed to efficiently determine the optimal parameter vector θ within predefined bounds. For FHN-SR and UBSR, θ=[h,g]; for classical SR, θ=[b,h] with a fixed to unity. In each iteration, every particle represents a candidate parameter set; the preprocessed input u(t) is fed into the SR model to produce the output y(t), and the fitness is defined as the SNR around the target characteristic frequency $f_{c}$, which is maximized as given in Equation (9).

(9) $${\mathrm{SNR}}_{\mathrm{dB}}=10\;\log_{10}\left(\frac{P_{\mathrm{sig}}}{P_{\mathrm{noise}}}\right)\;,\;P_{\mathrm{noise}}=P_{\mathrm{tot}}-P_{\mathrm{sig}}\;,$$

Here, $P_{sig}$ is computed by accumulating the one-sided spectral power within a narrow band around $f_{c}$ (optionally including $2f_{c}$ and $3f_{c}$), while $P_{tot}$ denotes the one-sided total spectral power, excluding the DC component. PSO updates particle velocities and positions by learning from the personal best (pbest) and the global best (gbest):

(10) $$v_{i}^{\left(t+1\right)}=w_{t}v_{i}^{\left(t\right)}+c_{1}r_{1}⊙\left(p_{i}-x_{i}^{\left(t\right)}\right)+c_{2}r_{2}⊙\left(g-x_{i}^{\left(t\right)}\right),\;\;x_{i}^{\left(t+1\right)}=x_{i}^{\left(t\right)}+v_{i}^{\left(t+1\right)},$$

where $c_{1}$ and $c_{2}$ are acceleration coefficients, $r_{1},r_{2}\in [0,1]$ are random vectors, and ⊙ denotes element-wise multiplication.

### 3.4. Parameter Search Strategy

Since the three stochastic resonance models considered in this study differ in both dynamical order and potential-function form, their parameter search strategies are not defined in an identical manner. Specifically, classical SR retains the conventional parameterization of the first-order bistable SR system, in which a is fixed to 1 and the potential parameter b, together with the numerical integration step h, is optimized. By contrast, UBSR and FHN-SR are both formulated within the same second-order underdamped framework. Therefore, after fixing the potential parameters a and b, only the parameters directly governing the dynamic response, namely h and g, are optimized. This treatment does not imply that the potential parameters are unimportant; rather, it avoids redundant coupling between the potential-scale parameters and the dynamic parameters, reduces the search dimensionality, and improves the stability and reproducibility of the particle swarm optimization procedure. The specific search settings for the three SR models are summarized in Table 1.

For the practical implementation of PSO, a unified optimization setting was adopted for all SR-based methods in order to ensure a fair comparison under the same optimization framework. The inertia weight and the cognitive/social learning factors were set to w=0.72 and $c_{1}=c_{2}=1.49$, respectively, because these values are close to the commonly used constriction-factor-equivalent parameter setting in the PSO literature (w≈0.729, $c_{1}=c_{2}\approx 1.49445$), which is widely reported to provide a good balance between global exploration, local exploitation, and convergence stability [37]. In addition, the particle velocity was limited to 20% of the corresponding search span in each dimension to avoid excessively large jumps and to improve numerical stability during iterative optimization. The swarm size and the maximum iteration number were set to 25 and 40, respectively, as a compromise between optimization reliability and computational cost, since each fitness evaluation requires repeated numerical integration of the SR model. The random seed was fixed to 1 only to ensure reproducibility of the optimization results.

The search ranges were selected according to the structural characteristics of the considered SR models together with numerical stability considerations. For classical SR, the parameter b was searched within $\left[0.1,10\right]$ so as to cover a sufficiently broad range of bistable potential shapes, while the integration step h was searched within $\left[0.002,0.1\right]$ to avoid excessively coarse discretization or unnecessarily high computational cost. For UBSR and FHN-SR, the damping coefficient g was searched within $\left[0.01,0.3\right]$, which covers weak-to-moderate damping conditions while avoiding over-damping that may suppress the resonance effect. For these two second-order models, the potential parameters were fixed to a=b=1, and only h and g were optimized. This choice follows the reduced-dimensional search strategy discussed above, which helps reduce redundant parameter coupling, improve optimization stability, and maintain a clear and comparable focus on the dynamic response of the models.

**Figure 4:**
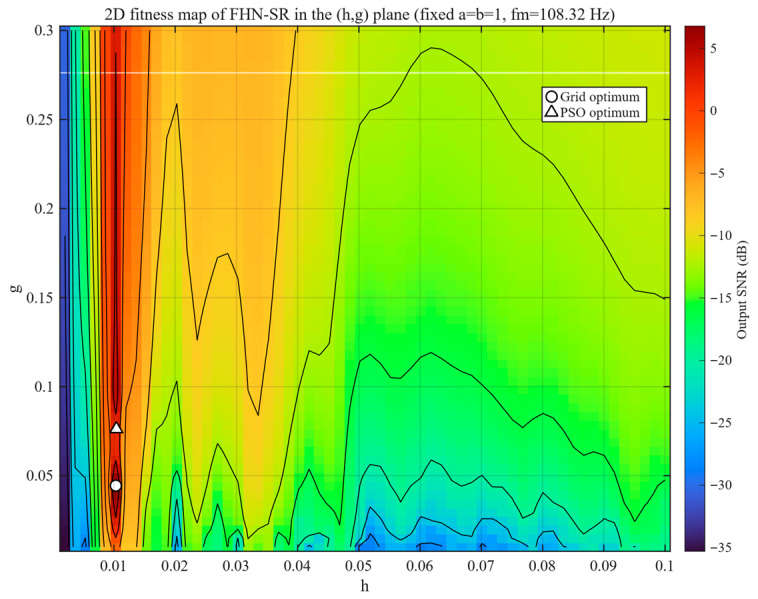
Two-dimensional fitness map of FHN-SR in the h-g plane with fixed a=b=1.

To further verify the rationality of this reduced-dimensional search strategy, a two-dimensional fitness scan of the h-g plane was performed for FHN-SR under the condition a=b=1, using the MCDS inner-race fault signal as an example. As shown in Figure 4, after fixing a=b=1, the fitness landscape of FHN-SR in the h-g plane exhibits a clear dominant high-fitness region rather than a scattered distribution of irregular local peaks. Both the grid optimum and the PSO optimum are located within the same high-fitness band, indicating that the major performance variation in the system can still be effectively captured through the joint adjustment of h and g after the potential parameters are fixed. Meanwhile, the landscape is more sensitive along the h-direction, whereas a relatively broader high-fitness range is observed along the g-direction. This result suggests that the two-parameter search preserves the key dynamic characteristics of the system while also improving the reliability of the optimization process.

Accordingly, the use of different parameter search strategies in this study is determined by the structural characteristics and comparative roles of the three SR models, and is therefore considered reasonable for the present experimental framework.

## 4. Experimental Verification

To verify the effectiveness of the proposed method in bearing fault feature enhancement, experimental investigations are carried out on multiple fault cases. In this section, FHN-SR is evaluated together with classical SR and UBSR under the same signal-processing framework, while a Fast-Kurtogram-based envelope-analysis method is additionally included as a conventional non-SR baseline. Through this design, the comparison is performed at two levels: first, within the SR family to examine the influence of different dynamical structures and potential forms; second, against a traditional demodulation-based method to assess the practical engineering value of the proposed approach.

This section validates the proposed method by using two bearing vibration datasets obtained from different experimental platforms. The inner-race and outer-race fault data were acquired using the MCDS, whereas the rolling element fault data were obtained from the CWRU bearing dataset [38].

### 4.1. Experimental Data and Fault Case Description

To verify the effectiveness of the proposed method under different fault conditions, two bearing fault datasets from different sources were employed in this study, namely the CWRU 48 kHz drive-end bearing dataset and the MCDS experimental platform. The rolling-element fault case was selected from the CWRU dataset, corresponding to file 123.mat, which contains a ball defect with a fault diameter of 0.007 in and a motor speed of 1772 rpm. The inner-race and outer-race fault cases were obtained from the MCDS platform using a 6205 deep-groove ball bearing, and the vibration signals were acquired at a sampling frequency of 51.2 kHz. In the MCDS dataset, the fault label encodes both the fault location and the defect severity; specifically, IR8 and OR8 denote the inner-race fault and outer-race fault, respectively, and the number 8 indicates that the circumferential extent of the defect region on the bearing raceway is 8°. The detailed defect locations, shapes, and sizes of the considered fault cases are summarized in Table 2.

### 4.2. Bearing Rolling Element Fault Diagnosis

**Figure 5:**
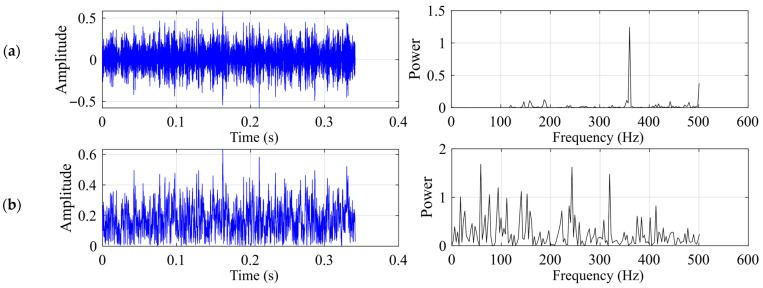
Input signal for the rolling element fault case: (**a**) time-domain waveform and frequency spectrum of the raw vibration signal; (**b**) time-domain waveform and envelope spectrum of the demodulated signal.

This subsection presents the experimental results for the rolling element fault. The motor speed is 1722 rpm, and the shaft rotational frequency is 28.7 Hz. The rolling element fault characteristic frequency *f*_c_ should be around 139.21 Hz. Figure 5 shows the time-domain waveform and frequency spectrum of the raw vibration signal, together with the time-domain waveform and envelope spectrum of the demodulated signal for the rolling element fault case.

**Figure 6:**
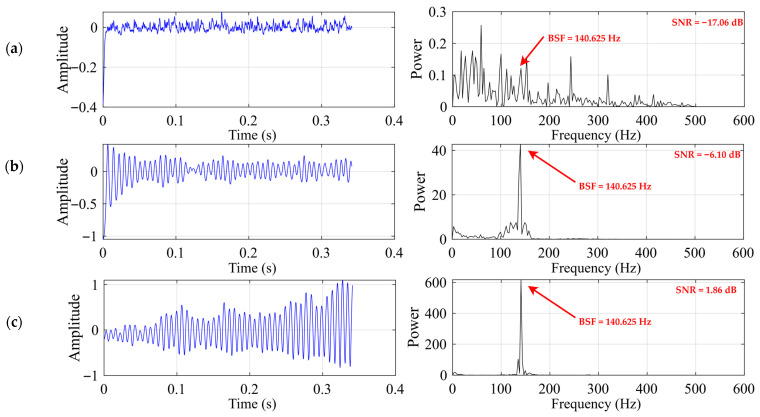
Output signal for the rolling element fault case: (**a**) classical SR (b = 10 h = 0.0592); (**b**) UBSR (h = 0.0121 g = 0.1208); (**c**) FHN-SR (h = 0.0149 g = 0.0195).

Figure 6 and Table 3 jointly present the comparative results of the three SR methods for the rolling element fault case. As shown in Figure 6, all three methods produce some degree of enhancement near the theoretical frequency, with the dominant peak located at the nearest spectral bin around 140.625 Hz, but their performances differ substantially. Classical SR exhibits only a weak spectral response, with the fault-related component still heavily buried in the background, leading to the lowest output SNR. UBSR improves the visibility of the Ball Spin Frequency (BSF) component and yields a clearer concentration of energy around the target frequency, indicating that the second-order underdamped bistable structure is more effective than the classical first-order model for this fault type. Among the three methods, FHN-SR provides the most distinct enhancement result, as reflected by the sharpest spectral peak at the BSF location, the highest output SNR, and the largest peak power in Table 3. These results indicate that, for the rolling element fault case, FHN-SR achieves the most effective extraction of the fault-related component, while UBSR shows intermediate performance and classical SR remains relatively limited.

**Figure 7:**
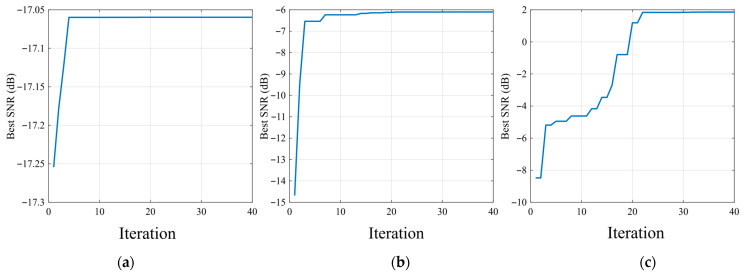
PSO convergence curves for the rolling element fault case: (**a**) classical SR; (**b**) UBSR; (**c**) FHN-SR.

As shown in Figure 7, the PSO convergence curves of the three SR methods exhibit distinct optimization behaviors for the rolling element fault case. Classical SR converges rapidly within the first few iterations, but its final best SNR remains at a very low level, indicating that its enhancement capability for this fault type is limited. Compared with classical SR, UBSR achieves a clear improvement during the iterative search and converges to a noticeably higher SNR, showing that the second-order underdamped bistable structure is more effective in capturing the rolling element fault-related component. Among the three methods, FHN-SR exhibits the most favorable convergence behavior, with a continuous increase in best SNR over a broader range of iterations and the highest final value. This result suggests that the FHN-SR parameter space is more conducive to effective optimization and is consistent with the corresponding output results, further confirming its superiority for the rolling element fault case.

**Figure 8:**
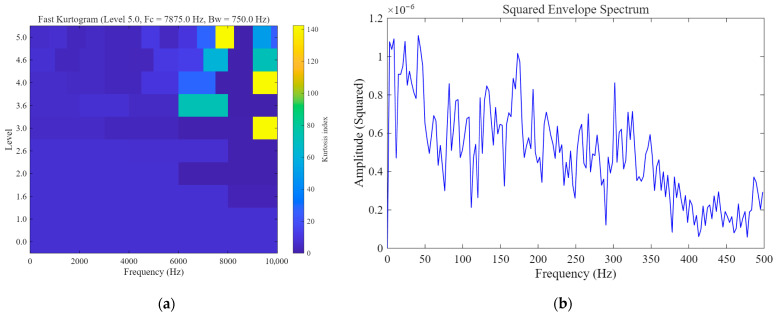
Fast-Kurtogram-based baseline result for the rolling element fault case: (**a**) Fast Kurtogram; (**b**) squared envelope spectrum obtained from the selected frequency band.

**Figure 9:**
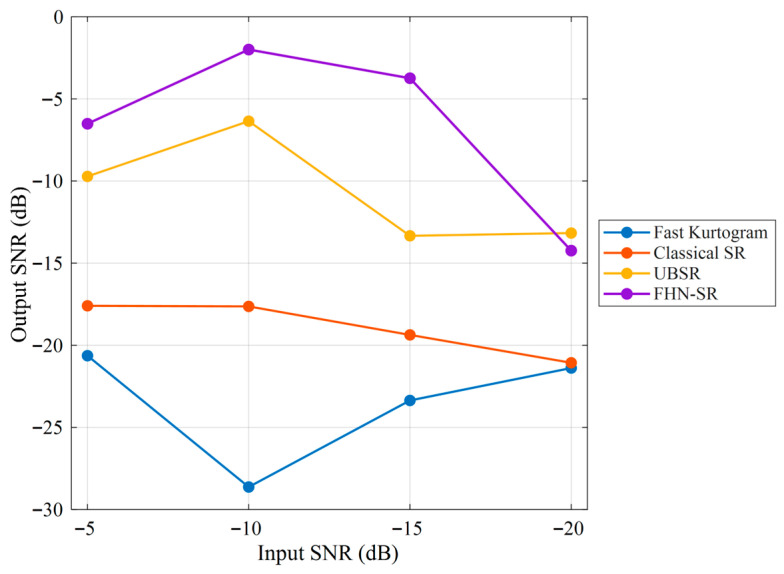
Quantitative noise-robustness comparison of different methods under the rolling element fault condition.

As shown in Figure 8, for the rolling element fault case, although Fast Kurtogram can identify an optimal frequency band, the squared envelope spectrum obtained from the selected band does not exhibit a clear and prominent peak near the theoretical BSF, and the fault-related energy remains rather dispersed. This indicates that the conventional envelope-analysis baseline has limited capability in extracting the weak fault-related component in this case. Furthermore, Figure 9 presents the quantitative robustness comparison of different methods under varying input SNR conditions. It can be observed that Fast Kurtogram consistently yields the lowest output SNR across the tested noise levels, indicating high sensitivity to strong noise interference. Classical SR maintains a generally low output level and shows limited enhancement capability, whereas UBSR outperforms Classical SR in most cases but still suffers a noticeable degradation as the noise becomes more severe. In contrast, FHN-SR achieves the highest output SNR under most tested conditions and remains competitive even in heavily contaminated environments, demonstrating that the proposed method provides not only superior weak-feature extraction for rolling element fault diagnosis but also stronger robustness against noise.

### 4.3. Bearing Inner-Race Fault Diagnosis

**Figure 10:**
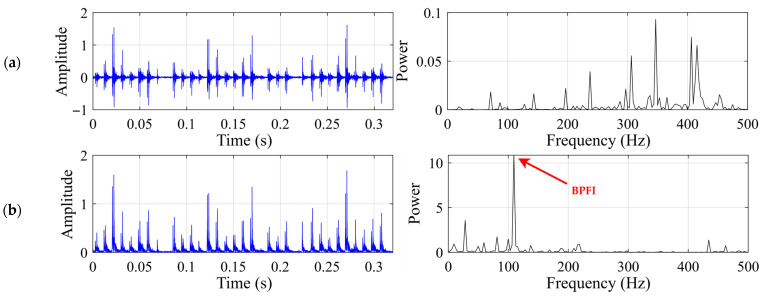
Input signal for the inner-race fault case: (**a**) time-domain waveform and frequency spectrum of the raw vibration signal; (**b**) time-domain waveform and envelope spectrum of the demodulated signal.

This subsection presents the experimental results for the inner-race fault. The motor speed is 1200 rpm, and the shaft rotational frequency is 20 Hz. The inner-race fault characteristic frequency *f*_c_ should be around 108.32 Hz. Figure 10 shows the time-domain waveform and frequency spectrum of the raw vibration signal, together with the time-domain waveform and envelope spectrum of the demodulated signal for the inner-race fault case.

**Figure 11:**
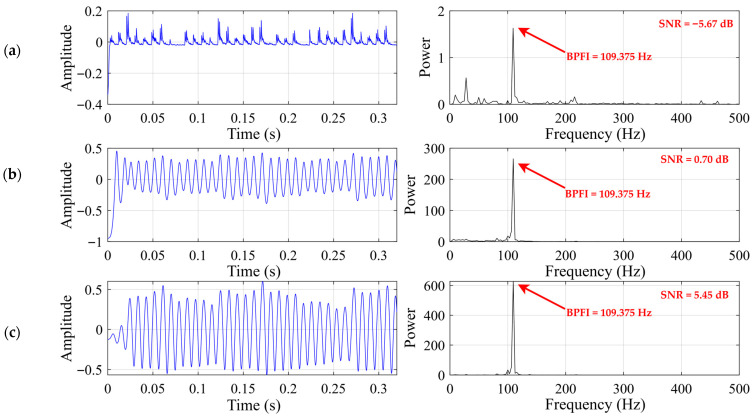
Output signal for the inner-race fault case: (**a**) classical SR (b = 10 h = 0.0341); (**b**) UBSR (h = 0.01 g = 0.1006); (**c**) FHN-SR (h = 0.0104 g = 0.0763).

As shown in Figure 11 and Table 4, all three SR-based methods are able to enhance the fault-related component near the theoretical inner-race characteristic frequency, but their enhancement performance differs markedly. In the classical SR result, although the Ball Pass Frequency of the Inner Race (BPFI) component at 109.375 Hz can be identified, the spectral peak remains relatively weak and the corresponding output SNR is still low, indicating limited suppression of background interference. After introducing the underdamped second-order bistable structure, UBSR produces a much more concentrated spectral response around the target frequency, and the output SNR is accordingly improved. By comparison, FHN-SR exhibits the most prominent enhancement effect, with the sharpest spectral peak at the BPFI location and the highest output SNR among the three methods. In the time domain, the FHN-SR output also shows a more regular oscillatory pattern than the other two methods, suggesting that the fault-related periodic component has been extracted more effectively. These results indicate that, for the inner-race fault case, FHN-SR provides superior feature enhancement capability over both classical SR and UBSR.

**Figure 12:**
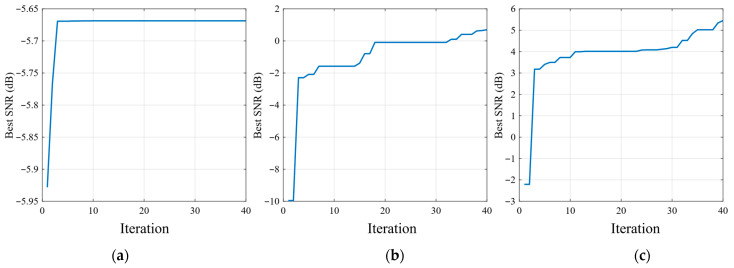
PSO convergence curves for the inner-race fault case: (**a**) classical SR; (**b**) UBSR; (**c**) FHN-SR.

Figure 12 shows that the PSO convergence behaviors of the three SR methods exhibit clear differences. The classical SR curve converges rapidly, but the final best SNR remains relatively low, indicating limited enhancement capability under the present fault condition. UBSR achieves a noticeable improvement over classical SR, with a higher final SNR and a more progressive optimization process. By comparison, FHN-SR reaches the highest best SNR among the three methods and maintains a stable upward trend during the iterative search, which suggests that the corresponding parameter space is more favorable for effective optimization. These convergence results are consistent with the output spectra shown previously and further support the superiority of FHN-SR in the inner-race fault case.

**Figure 13:**
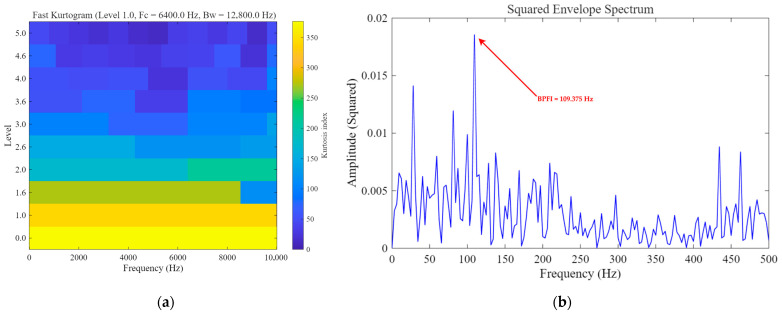
Fast-Kurtogram-based baseline result for the inner-race fault case: (**a**) Fast Kurtogram; (**b**) squared envelope spectrum obtained from the selected frequency band.

To further evaluate the effectiveness of the proposed method from an engineering perspective, a Fast-Kurtogram-based envelope-analysis scheme was additionally employed as a conventional non-SR baseline for comparison. Figure 13 shows the Fast-Kurtogram-based result for the inner-race fault case. The selected band yields a visible peak near the theoretical characteristic frequency BPFI =  109.375 Hz, indicating that the fault-related component can be extracted to some extent. However, compared with the SR-based results, the squared envelope spectrum obtained by the Fast Kurtogram still exhibits a relatively high spectral background and a less concentrated characteristic peak. In particular, the enhancement effect is weaker than that achieved by UBSR and FHN-SR, whose output spectra show clearer energy concentration around the target frequency. This comparison indicates that, although the Fast Kurtogram provides a meaningful conventional baseline, its fault-feature enhancement capability remains limited under the present inner-race fault condition.

**Figure 14:**
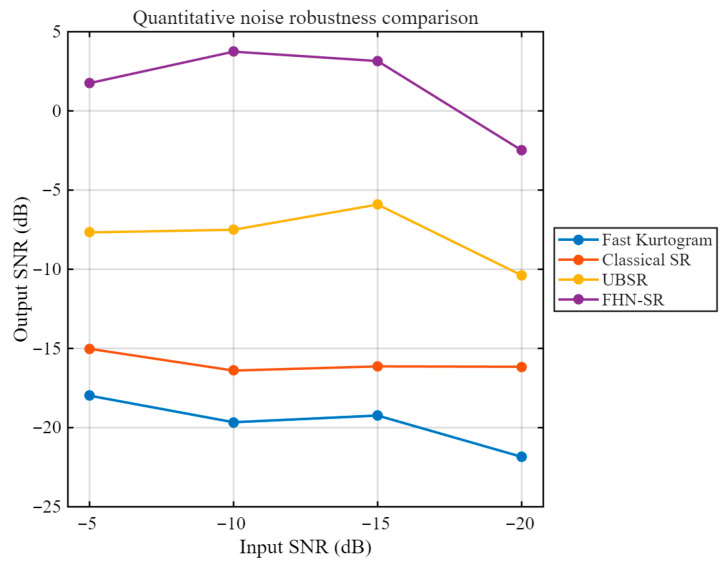
Quantitative noise-robustness comparison of different methods under the inner-race fault condition.

Figure 14 compares the output SNRs of different methods under progressively degraded input SNR conditions. It can be observed that FHN-SR consistently achieves the highest output SNR over the entire tested noise range, indicating a stronger noise-robust feature enhancement capability than the other methods. Although the output performance of all methods decreases to some extent as the input noise level becomes more severe, FHN-SR still maintains a clear advantage over classical SR, UBSR, and the Fast-Kurtogram-based baseline. In particular, the superiority of FHN-SR remains evident even at −20 dB input SNR, which demonstrates that the proposed method is more effective in preserving fault-related information under strong noise interference.

### 4.4. Bearing Outer-Race Fault Diagnosis

**Figure 15:**
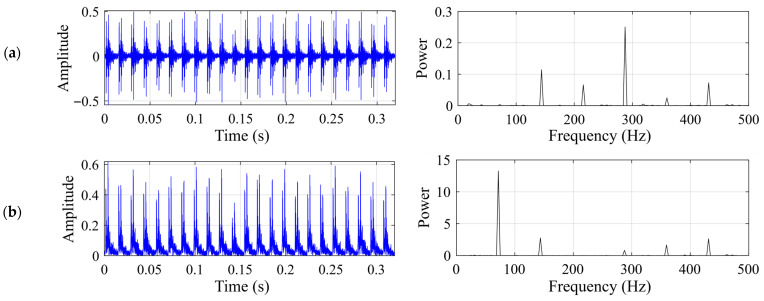
Input signal for the outer-race fault case: (**a**) time-domain waveform and frequency spectrum of the raw vibration signal; (**b**) time-domain waveform and envelope spectrum of the demodulated signal.

This subsection presents the experimental results for the outer-race fault. The motor speed is 1200 rpm, and the shaft rotational frequency is 20 Hz. The outer-race fault characteristic frequency *f*_c_ should be around 71.68 Hz. Figure 15 shows the time-domain waveform and frequency spectrum of the raw vibration signal, together with the time-domain waveform and envelope spectrum of the demodulated signal for the outer-race fault case.

**Figure 16:**
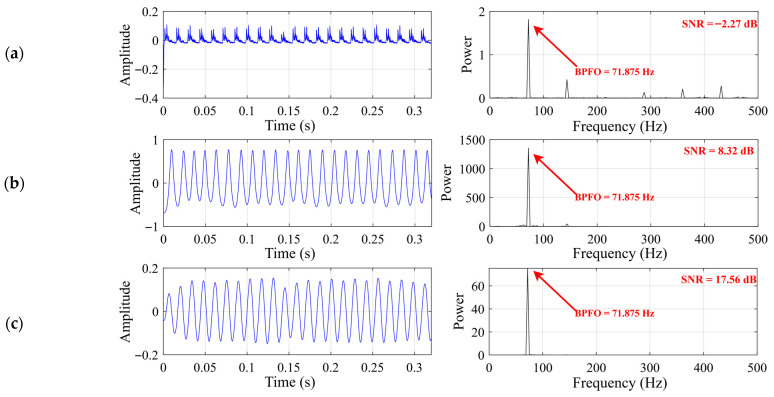
Output signal for the outer-race fault case: (**a**) classical SR (b = 10 h = 0.1); (**b**) UBSR (h = 0.0086 g = 0.0534); (**c**) FHN-SR (h = 0.007 g = 0.3).

Figure 16 and Table 5 jointly present the comparative results of the three SR methods for the outer-race fault case. As shown in Figure 16, all three SR-based methods are able to enhance the fault-related component at the theoretical outer-race characteristic frequency (approximately 71.875 Hz), but their enhancement performances differ markedly. Classical SR yields only a weak spectral response and remains at a relatively low output SNR. By contrast, both UBSR and FHN-SR produce much clearer spectral concentration around the target frequency, with substantially improved output SNRs. The quantitative results in Table 5 further support this tendency. Although UBSR exhibits the largest peak power at the Ball Pass Frequency of the Outer Race (BPFO) location, FHN-SR achieves the highest output SNR, indicating a more favorable balance between characteristic enhancement and background suppression. In other words, the superior performance of FHN-SR is reflected not simply in the absolute peak magnitude, but more importantly in its ability to concentrate fault-related energy while reducing spectral interference. These results demonstrate that, for the outer-race fault case, FHN-SR provides the most favorable overall enhancement effect among the compared SR methods.

**Figure 17:**
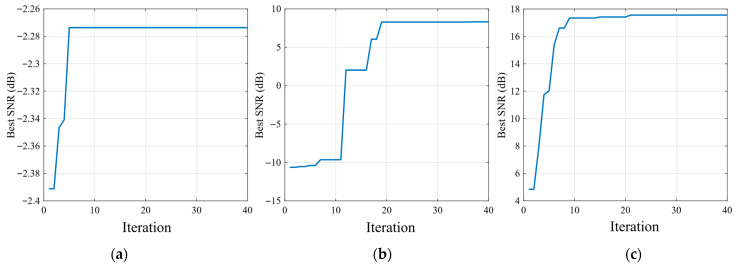
PSO convergence curves for the outer-race fault case: (**a**) classical SR; (**b**) UBSR; (**c**) FHN-SR.

As shown in Figure 17, the PSO convergence curves of the three SR methods exhibit clearly different optimization behaviors under the outer-race fault condition. Classical SR converges rapidly within the early iterations, but its final best SNR remains at a relatively low level, indicating limited enhancement capability for the present signal. Compared with classical SR, UBSR shows a more substantial improvement during the iterative search and reaches a markedly higher final SNR, suggesting that the second-order underdamped bistable structure is more effective for extracting the outer-race fault-related component. Among the three methods, FHN-SR attains the highest best SNR and exhibits the most favorable convergence trend, with a rapid increase in the early stage followed by stable convergence. This result indicates that the parameter space of FHN-SR is more amenable to effective optimization and is consistent with the corresponding output results, further confirming its superior feature-enhancement performance for the outer-race fault case.

**Figure 18:**
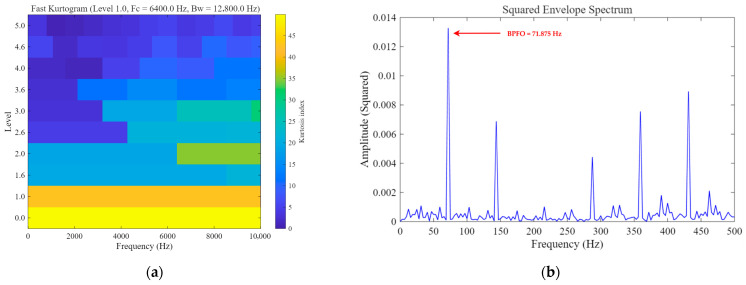
Fast-Kurtogram-based baseline result for the outer-race fault case: (**a**) Fast Kurtogram; (**b**) squared envelope spectrum obtained from the selected frequency band.

**Figure 19:**
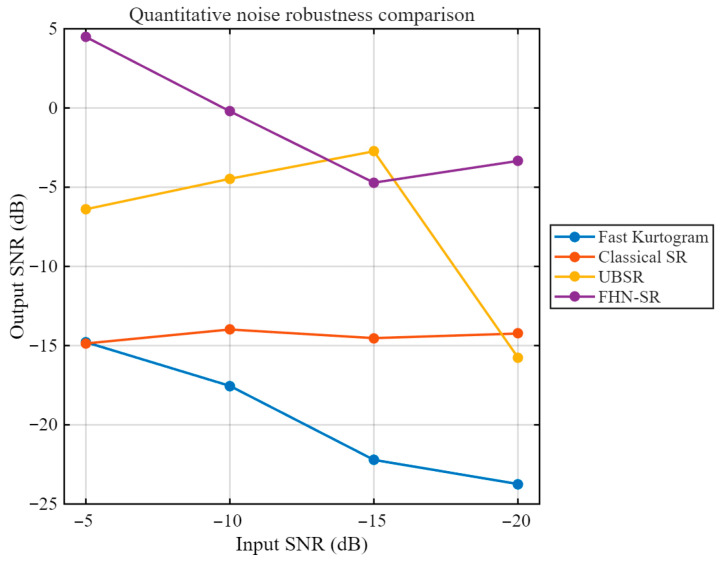
Quantitative noise-robustness comparison of different methods under the outer-race fault condition.

As shown in Figure 18, the Fast-Kurtogram-based method is able to identify the fault-related component at the theoretical outer-race characteristic frequency BPFO = 71.875 Hz, indicating that it can serve as a meaningful conventional baseline. However, the squared envelope spectrum still contains a relatively high background level, suggesting that its feature-enhancement capability remains limited. Figure 19 illustrates how the output performance of different methods changes as the noise level progressively increases. A clear separation can be observed among the four curves. The Fast-Kurtogram-based baseline and classical SR remain at relatively low output SNR levels throughout the tested range, whereas UBSR and FHN-SR show markedly stronger resistance to noise contamination. Although UBSR performs competitively at moderate noise levels, FHN-SR exhibits a more favorable overall trend, especially under the most severe noise condition. At −20 dB input SNR, FHN-SR still preserves a distinct advantage over the other methods, indicating that it is more capable of retaining the fault-related component when the background interference becomes dominant. This result further confirms the robustness of FHN-SR for the outer-race fault case.

### 4.5. Discussion on Computational Efficiency and Practical Applicability

Although the present study mainly focuses on the fault-feature enhancement performance of the proposed method, its computational efficiency should also be discussed from a practical viewpoint. The major computational burden of the PSO-based FHN-SR framework arises from the repeated numerical integration of the stochastic resonance model during parameter optimization, since a complete fitness evaluation must be performed for each particle in each iteration. Compared with conventional single-pass signal-processing methods, the proposed framework is therefore more computationally demanding. However, this additional cost is compensated for by the improved robustness and adaptivity of weak-feature extraction obtained through data-driven parameter optimization.

From the viewpoint of practical deployment, the current framework is more suitable for offline diagnosis or quasi-online analysis. Since the fitness evaluations of different particles are mutually independent, the proposed framework also has good potential for parallel implementation on multi-core CPUs, GPUs, or other high-performance computing platforms. It should be noted that strict real-time fault monitoring has not been specifically validated in the present study. Therefore, at the current stage, the proposed method is more appropriately regarded as an effective offline weak-feature enhancement and fault-diagnosis tool. Further work toward real-time application would require additional efforts in optimization simplification, search-space reduction, parallel acceleration, and hardware-oriented implementation.

## 5. Conclusions

This study proposed a PSO-based FHN-SR method for bearing fault-feature enhancement and validated it using the rolling-element fault case from the CWRU dataset as well as the inner-race and outer-race fault cases from the MCDS platform. Comparative results against classical SR, UBSR, and a Fast-Kurtogram-based baseline demonstrate that the proposed FHN-SR method achieves clearer spectral energy concentration around the fault-related frequency and higher output SNR. In particular, the method maintains favorable robustness under degraded noise conditions. These results indicate that, within the same second-order underdamped framework, the FHN-type potential provides a more effective balance between fault-feature enhancement and background suppression, making it more suitable for weak-fault-feature extraction under complex noise environments.

In general, the proposed method offers several advantages, including strong weak-feature enhancement capability, good noise robustness, and reduced dependence on manual parameter tuning through PSO-based adaptive optimization. At the same time, some limitations should also be acknowledged, such as the additional computational burden introduced by iterative parameter optimization, the still limited range of validation conditions considered in the present study, and the need for further investigation regarding its applicability to strict real-time scenarios. Future work will therefore focus on broader experimental validation, computational efficiency improvement, and further development toward practical engineering applications.

## Figures and Tables

**Table 1:** Parameter search settings and dynamical frameworks of the three SR methods.

Method	Fixed	Optimized	Framework
Classical SR	a = 1	b, h	first-order bistable
UBSR	a = 1, b = 1	g, h	underdamped second-order
FHN-SR	a = 1, b = 1	g, h	underdamped second-order

**Table 2:** Summary of defect information for different bearing fault cases.

Fault Case	Data Source	Fault Location	Defect Shape	Defect Size
Rolling-element fault	CWRU 48 kHz drive-end dataset	rolling element	Localized point-like defect	0.007 in diameter
Inner-race fault	MCDS platform	Inner race	Localized rectangular raceway defect	8° circumferential defect extent
Outer-race fault	MCDS platform	Outer race	Localized rectangular raceway defect	8° circumferential defect extent

**Table 3:** Quantitative comparison of different SR methods for the rolling element fault case.

Methods	Classical SR	UBSR	FHN-SR
SNR	−17.06	−6.10	1.86
Peak power at BSF	0.121	42.785	617.373

**Table 4:** Quantitative comparison of different SR methods for the inner-race fault case.

Methods	Classical SR	UBSR	FHN-SR
SNR	−5.67	0.70	5.45
Peak power at BPFI	1.63	226.024	627.494

**Table 5:** Quantitative comparison of different SR methods for the outer-race fault case.

Methods	Classical SR	UBSR	FHN-SR
SNR	−2.27	8.32	17.56
Peak power at BPFO	1.814	1356.67	75.347

## Data Availability

Publicly available datasets were analyzed in this study. The public data are available from the Case Western Reserve University (CWRU) Bearing Data Center. The experimental data generated during the current study from the authors’ test rig are available from the corresponding author upon reasonable request.
